# Predictive modelling of level IIb lymph node metastasis in oral squamous cell carcinoma

**DOI:** 10.1038/s41598-021-96827-1

**Published:** 2021-09-02

**Authors:** Hyunwoo Yang, Nak-Hoon Son, Sung Hwa Lee, Dongwook Kim, Hyung Jun Kim, In-ho Cha, Woong Nam

**Affiliations:** 1 Department of Oral and Maxillofacial Surgery, Yonsei University College of Dentistry, 50-1 Yonsei-ro, Seodaemun-gu, Seoul, 03722 Republic of Korea; 2 Division of Biostatistics, Yongin Severance Hospital, Yonsei University College of Medicine, Yongin, 16995 Gyeonggi-do Republic of Korea

**Keywords:** Cancer, Diseases, Medical research, Oncology, Risk factors

## Abstract

The aim of the present study was to examine the conditions, characteristics, and risk factors of level IIb lymph node metastases in oral squamous cell carcinoma and to formulate surgical criteria for level IIb lymph node dissection. We analyzed clinical and pathological records for 541 oral squamous carcinoma patients in relation to level IIb metastasis. Univariate and multivariate analyses were performed to detect risk factors for level IIb lymph node metastasis; a predictive model was built based on multivariate analysis and tested in a validation group. Univariate and multivariate analyses using the training group indicated that level IIa metastasis and Lymphovascular permeation (LVP) were two independent risk factors for level IIb lymph node metastasis. This model was built and tested in a validation group, the area under the curve being 0.697 (P < .0.001). The model’s sensitivity was 66.7% and specificity was 77.4%. Nomogram incorporating validated variables was developed for level IIb metastasis prediction. Expected survival probabilites were analysed to specify significance of model's variable on patients’ overall survival and recurrence. Level IIb dissection should be performed in patients with level IIa metastasis and LVP. However, thorough consideration of the oncologic safety of omitting level IIb dissection is compulsory.

## Introduction

Oral cancer is a global problem, with over 350,000 new patients each year and more than 170,000 deaths^1^. Paradoxically, a majority of cases are detected at an advanced stage, slightly more than half of patients surviving 5 years despite a well-defined tumor progression model and relatively amenable examination^2^. Cervical lymph node metastases in oral squamous cell carcinoma (OSCC), which account for more than 90% of oral malignancies, are common. The presence of lymph node metastasis is one of the most adverse prognostic factors in OSCC^3^. It has been reported clinically evident in approximately 40% of patients, with occult disease rates ranging from 15 to 34%^4,5^. Consequently, OSCC is often treated with simultaneous neck dissection for either therapeutic, diagnostic, or prophylactic purposes. After Virchow first formulated the definition and emphasized the metastatic trend of malignant lesions in 1885 through "Cellular pathology", neck dissection as currently conceived emerged^6,7^. Later, radical neck dissection was introduced, becoming the standard in managing oral cancer metastatic lymph node^8^. However, recognizing that morbidity was clearly associated with the increasing extent of neck dissections, surgeons consistently sought to decrease the extent to achieve a balance between oncologic safety and acceptable morbidity^9^. Due to their invasive nature, radical neck dissections are now performed only for patients with large metastatic nodes with extranodal extension into the non-lymphatic structures^10^. In line with such atmosphere, modified radical neck dissection (MRND) or functional neck dissection (FND), which preserves major anatomical structures while dissecting lymphatic group was introduced and performed^11^. There is also an overall consensus for performing selective neck dissections (SND) rather than modified radical neck dissections (MRND) for patients with clinical node-negative necks^12^. In spite of such gradual establishment of protocol for treating neck in OSCC patients, there is an ongoing debate concerning dissection of level IIB in both node-negative and positive patients, primarily due to the low incidence of metastasis in this region and possible injury to the spinal accessory nerve (SAN)^9^. Previous studies have argued for and against dissecting level IIB based on descriptive results regarding the level IIb lymph node metastasis rate^13,14^. However, to our knowledge, no predictive model has been generated to assist decision on extending dissection of level IIb compartment based on a OSCC patient cohort. In this study, we conducted a multi-spectral analysis of the frequency and pattern of level IIb lymph node metastases, detected risk factors, and built a predictive model based on these risk factors with a large OSCC patient cohort.

## Results

### Clinicopathologic characteristics

Of the 359 patients whose records were suitable for analysis, 139 were in early stages (I-II) and 220 patients in developed stages (III-IV) according to the American Joint Committee on Cancer (AJCC) 8th edition Cancer Staging Manual. Among them, 12 patients had level IIb metastasis with three isolated metastases. Table 1 presents the clinical characteristics of the dataset.

**Table 1:** Baseline characteristics.

Variable	N	Total
**Sex**	359	
Male		235 (65.5%)
Female		124 (34.5%)
**Ages**	359	59.3 ± 11.7
**TNM stage**^**a**^	359	
Early (I–II)		139 (38.7%)
Developed (III–IV)		220 (61.3%)
**pN**	359	
N0		212 (59.1%)
N +		147 (40.9%)
**cT**	359	
Early (T1–T2)		52 (14.5%)
Developed (T3–T4)		307 (85.5%)
**cN**	359	
N0		207 (57.7%)
N +		152 (42.3%)
**Site**	359	
Buccal cheek		36 (10.0%)
Floor of mouth		25 (7.0%)
Lip		2 (0.6%)
Mandible		120 (33.4%)
Maxilla		45 (12.5%)
Retromolar trigone		36 (10.0%)
Tongue		95 (26.5%)
**Resection margins**	359	
Free		252 (70.2%)
Dysplasia		25 (7.0%)
Presence		82 (22.8%)
**Histologic grade**	343	
Well differentiated		53 (15.5%)
Moderately differentiated		233 (67.9%)
Poorly differentiated		57 (16.6%)
**Bone marrow invasion**	160	
No		22 (13.8%)
Yes		138 (86.2%)
**Perineural invasion**	225	
No		187 (83.1%)
Yes		38 (16.9%)
**Lymphovascular permeation**	223	
No		186 (83.4%)
Yes		37 (16.6%)
**Depth of invasion**	77	8.0 [6.0–15.0]
**Extra nodal extension**	141	
Yes		50 (35.5%)
No		91 (64.5%)
**Neck levels of metastatic lymph node**		
Ia		11 (3.1%)
Ib		63 (17.5%)
IIa		46 (12.8%)
IIb		12 (3.3%)
III		20 (5.6%)
IV		6 (1.7%)
V		4 (1.1%)

Data were reported as mean ± standard deviation (SD) or median [Q1–Q3] for continuous variables and n (%) for categorical variables.

^a^According to AJCC 8th classification.

*pN* pathologic N, *cT* clinical T, *cN* clinical N.

All 359 OSCC patients who underwent neck dissection were split randomly into two mutually exclusive groups: a training group of 224 patients to detect risk factors for level IIb lymph node metastasis and build a predictive model and a validation group of 135 patients to verify the model. There were no statistically significant differences in variables between the two sets (Table 2).

**Table 2:** Baseline characteristics, training and validation groups.

Variable	Training	Validation	p value
	(N = 224)	(N = 135)	
**Sex**			0.375
Male	151 (67.4%)	84 (62.2%)	
Female	73 (32.6%)	51 (37.8%)	
**Age**	59.0 ± 11.6	59.8 ± 12.0	0.531
**TNM stage**			0.863
Early (T1–T2)	88 (39.3%)	51 (37.8%)	
Developed (T3–T4)	136 (60.7%)	84 (62.2%)	
**pN**			0.787
N0	134 (59.8%)	78 (57.8%)	
N +	90 (40.2%)	57 (42.2%)	
**cT**			0.222
Early (T1–T2)	28 (12.5%)	24 (17.8%)	
Developed (T3–T4)	196 (87.5%)	111 (82.2%)	
**cN**			1.000
N0	129 (57.6%)	78 (57.8%)	
N +	95 (42.4%)	57 (42.2%)	
**Site**			0.889
Buccal cheek	24 (10.7%)	12 (8.9%)	
Floor of mouth	14 (6.2%)	11 (8.1%)	
Lip	2 (0.9%)	0 (0.0%)	
Mandible	71 (31.7%)	49 (36.3%)	
Maxilla	28 (12.5%)	17 (12.6%)	
Retromolar trigone	24 (10.7%)	12 (8.9%)	
Tongue	61 (27.2%)	34 (25.2%)	
**Resection margins**			0.066
Free	152 (67.9%)	100 (74.1%)	
Dysplasia	21 (9.4%)	4 (3.0%)	
Presence	51 (22.8%)	31 (23.0%)	
**Histologic grade**			0.246
Well	32 (15.0%)	21 (16.2%)	
Moderate	140 (65.7%)	93 (71.5%)	
Poor	41 (19.2%)	16 (12.3%)	
**Bone marrow invasion**			0.352
No	11 (11.2%)	11 (17.7%)	
Yes	87 (88.8%)	51 (82.3%)	
**Perineural invasion**			0.542
No	121 (84.6%)	66 (80.5%)	
Yes	22 (15.4%)	16 (19.5%)	
**Lymphovascular permeation**			0.374
No	118 (85.5%)	68 (80.0%)	
Yes	20 (14.5%)	17 (20.0%)	
**Depth of invasion**	8.0 [ 6.0—13.0]	8.0 [ 7.0—15.0]	0.715
**Extra-nodal extension**			0.238
Yes	26 (31.0%)	24 (42.1%)	
No	58 (69.0%)	33 (57.9%)	

Data were reported as mean ± standard deviation (SD) or median [Q1–Q3] for continuous variables and n (%) for categorical variables. P values were calculated by Student’s t-test or Mann–Whitney U test for continuous variables and chi-square test or Fisher's exact test for categorical variables.

*pN* pathologic N, *cT* clinical T, *cN* clinical N.

### Risk factors for level IIb lymph node metastasis by univariate and multivariate analyses

To investigate risk factors for level IIb lymph node metastasis, 20 categorical and numerical variables were included (Table 3) in a univariate analysis of the training group (n = 224). The following factors were associated with level IIb lymph node metastasis: LVP (OR = 10.24; p = 0.014), level IIa metastasis (OR = 9.80; p = 0.004), level III metastasis (OR = 11.77; p = 0.003), level IV metastasis (OR = 17.92, p = 0.026), and level V metastasis (OR = 17.92, p = 0.026).

**Table 3:** Logistic regression for IIb metastasis in the training group. OR, odds ratio; CI, confidence interval.

Variable	Univariable	
	OR (95% CI)	p value
**Sex**		
Male	1	
Female	0.82 (0.12, 3.92)	0.818
**Age**	1.00 (0.94, 1.07)	0.945
**TNM stage**		
Early (I–II)	1	
Developed (III–IV)	1.64 (0.35, 11.64)	0.559
**pN**		
N0	1	
N +	195,904,539 (0.00, Inf)	0.994
**cT**		
Early (T1–T2)	1	
Developed (T3–T4)	0.85 (0.19, 16.43)	0.885
**cN**		
N0	1	
N +	3.53 (0.74, 25.02)	0.137
**Site**		
Buccal cheek	1	
Floor of mouth	1.00 (0.00, Inf)	1.000
Lip	1.00 (0.00, Inf)	1.000
Mandible	9,112,058 (0.00, Inf)	0.994
Maxilla	11,643,185 (0.00, Inf)	0.994
Retromolar trigone	28,578,726 (0.00, Inf)	0.994
Tongue	10,656,474 (0.00, Inf)	0.994
**Resection margins**		
Free	1	
Dysplasia	0.00(0.00, Inf)	0.996
Presence	0.00 (0.00, Inf)	0.994
**Histologic grade**		
Well	1	
Moderate	0.68 (0.08, 13.97)	0.741
Poor	2.45 (0.30, 50.82)	0.448
**Bone marrow invasion**		
No	1	
Yes	0.48 (0.06, 9.92)	0.532
**Perineural invasion**		
No	1	
Yes	0.00 (0.00, Inf)	0.994
**Lymphovascular permeation**		
No	1	
Yes	10.24 (1.59, 82.10)	**0.014**
**Depth of invasion**	0.84 (0.40, 1.18)	0.523
**Extra-nodal extension**		
Yes	1	
No	2.89 (0.46, 56.03)	0.339
**Ia**		
No	1	
Yes	7.07 (0.34, 54.12)	0.095
**Ib**		
No	1	
Yes	0.90 (0.05, 5.48)	0.921
**IIa**		
No	1	
Yes	9.80 (2.05, 52.10)	**0.004**
**III**		
No	1	
Yes	11.77 (2.14, 59.11)	**0.003**
**IV**		
No	1	
Yes	17.92 (0.77, 214.95)	**0.026**
**V**		
No	1	
Yes	17.92 (0.77, 214.95)	**0.026**

*pN* pathologic N, *cT* clinical T, *cN* clinical N.

Statistically significant variables from the univariate analysis were included in the multivariate analysis. As shown in Table 4, predictors of level IIb lymph node metastasis included LVP with an OR (95% confidence interval [CI]) of 7.59 (1.01, 68.29) and level IIa metastasis with an OR (95% CI) of 14.25 (1.91, 128.42).

**Table 4:** Logistic regression for IIb metastasis in the training group.

Variable	Multivariable	
	OR (95% CI)	p value
**Lymphovascular permeation**		
No	1	
Yes	7.59 (1.01, 68.29)	**0.047**
**IIa**		
No	1	
Yes	14.25 (1.91, 128.42)	**0.009**

*OR* odds ratio, *CI* confidence interval.

### Predictive model and nomogram for level IIb lymph node metastasis

**Figure 1:**
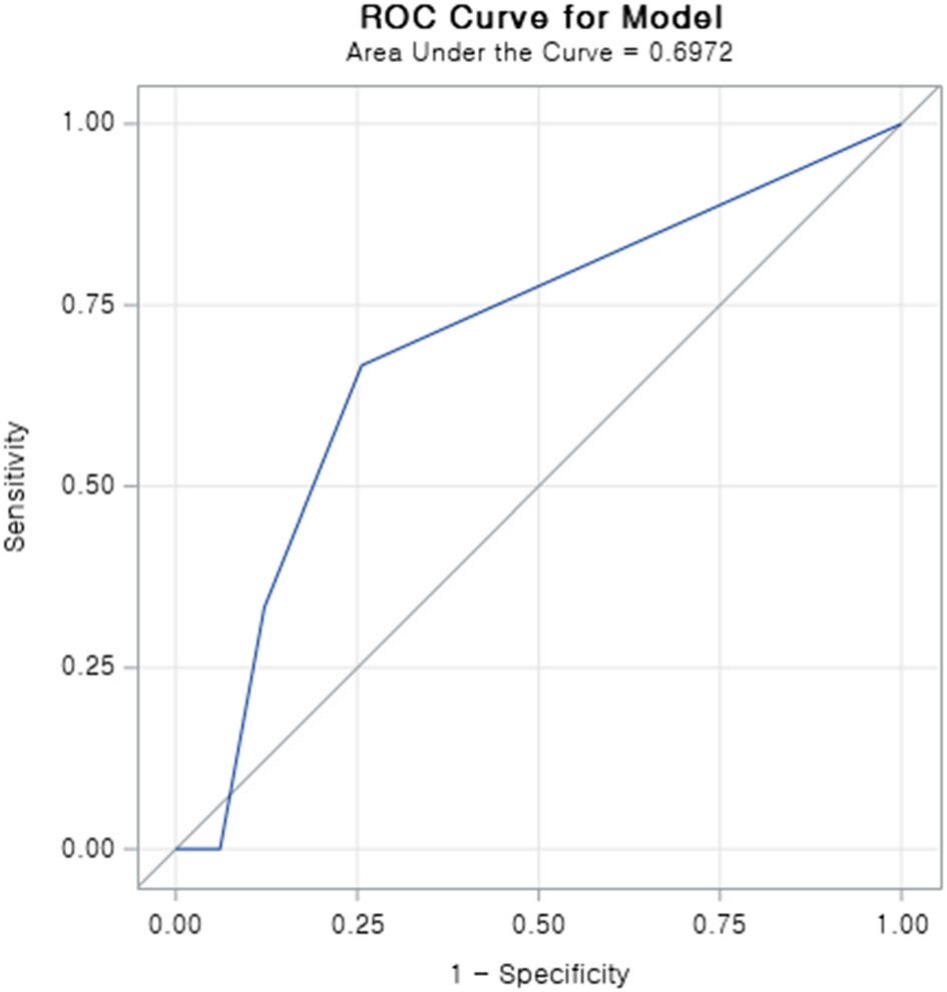
Receiver operating characteristic curve based on the level IIb score to predict level IIb lymph node metastasis (n = 135, AUC: 0.697, P < 0.001).

**Figure 2:**
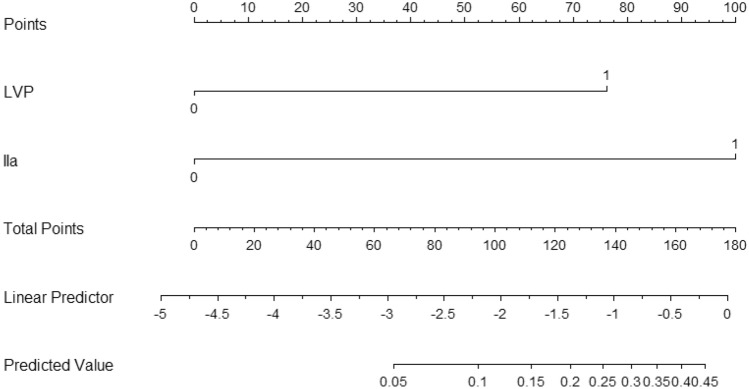
Prognostic nomogram for level IIb metastasis.

Based on multivariate analysis, we built a model to predict level IIb lymph node metastasis. The equation was as follows: logit[P]=-4.708+2.026×LVP+2.656×Level IIa metastasis. Receiver operating curve for the validation group is depicted in Fig. 1. The area under the curve (AUC) was 0.697, and the P value was less than 0.001. Cut-off value of this model was determined by the Youden index (the maximum potential effectiveness of a biomarker). Based on the cutoff value, the sensitivity was 66.7% and specificity 74.4%, with a model accuracy of 74.1%. To promote clinical applicability and increase user-friendliness, estimation nomogram for level IIb metastasis was developed utilizing variables(LVP, level IIa) obtained from Cox proportional hazard model (Fig. 2). Each variable in the model corresponds to a score, the sum of each score becomes a total score. The total scores ranges from 0 to 180, with corresponding metastasis risk of 0.05 to 0.45. The higher the score, the higher risk of level IIb metastasis.

### Survival analysis of OSCC patients according to follow up time

**Figure 3:**
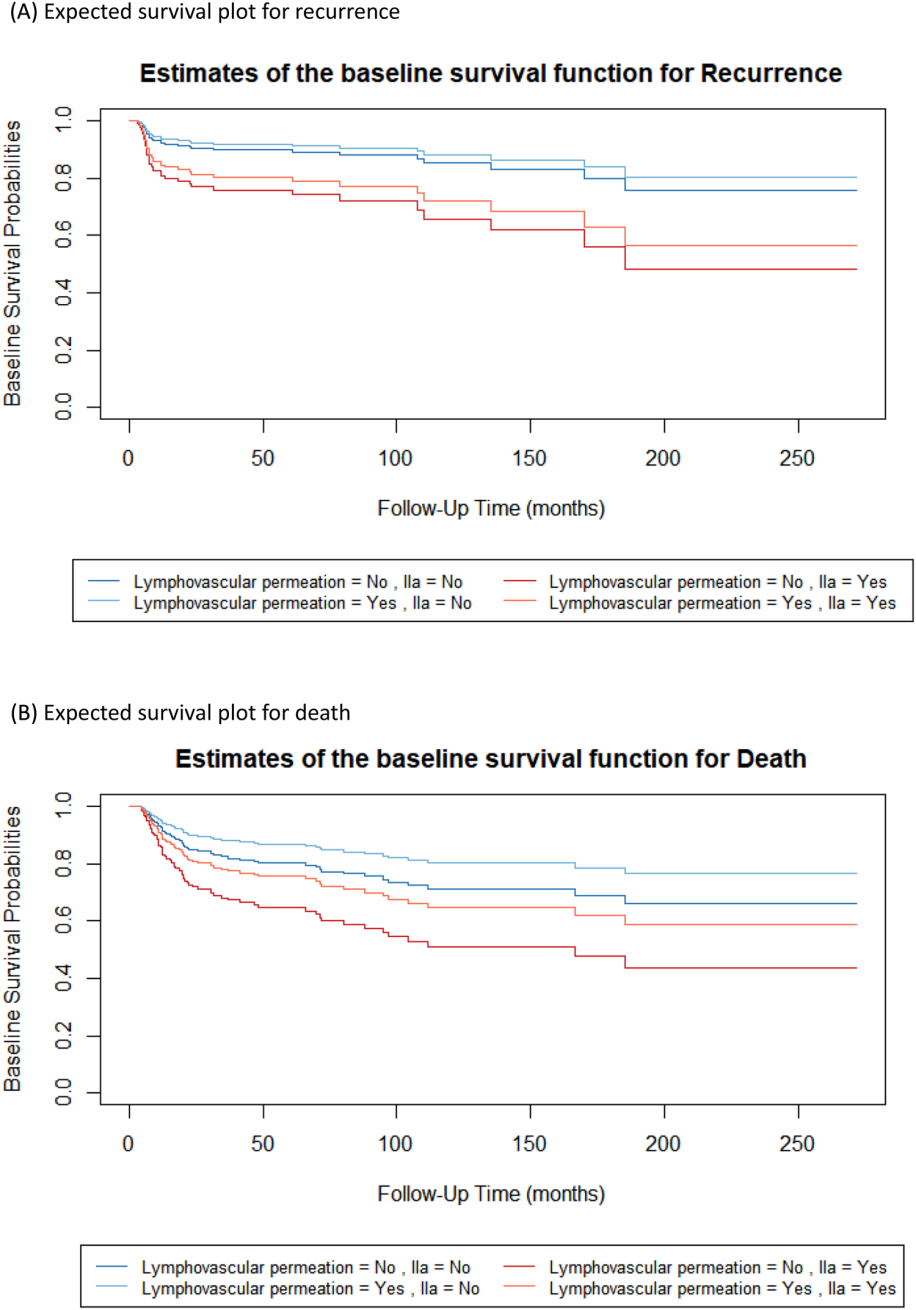
Survival analysis of OSCC(oral squamous cell carcinoma) patients of present study grouped into four categories : LVP positive/Level IIa negative, LVP negative/Level IIa negative, LVP negative/Level IIa positive, and LVP positive/Level IIa positive. **(A)** Expected survival probability plot for recurrence, **(B)** expected survival plot for death.

Extending the justification for LVP and level IIa, expected survival probabilities were analysed in the context of recurrence and overall death. Based on cox proportional hazard regression, hazard ratios of LVP and Level IIa were combined into following four cases : LVP positive/Level IIa negative, LVP negative/Level IIa negative, LVP negative/Level IIa positive, and LVP positive/Level IIa positive(Fig. 3). Survival plots revealed Level IIa metastasis had relatively stronger impact on recurrence and overall survival.

## Discussion

Many previous studies have made it clear that the pattern of cervical nodal spread in OSCC patients follows a somewhat predictable pathway^16–18^. Subsequently, extent of neck dissection, which primarily depends on the pattern and tumor subsite, has gradually become circumscribed to cervical levels I, II and III even in node-positive cases^19^. Cervical level II, however, remains one of the most prevalent regions of OSCC metastasis, requiring meticulous dissection^20^. Level IIb, referred to as “submuscular recess,” however, remains an area of controversy. The SAN itself serves as an anatomical border splitting level II into sublevels IIa and IIb^15^. To adequately remove the level IIb lymph node group, bounded anteromedially by the SAN, superiorly by the skull base, inferiorly by imaginary plane defined by the hyoid bone, and posteriorly by the posterior border of the sternocleidomastoid muscle, traction and elevation of the SAN is often inevitable. This may lead to significant shoulder dysfunction and discomfort. Despite the recent trend toward conservative surgical methods, some degree of shoulder morbidity has been reported even with intact SAN after neck dissection^21,22^. For this reason, and because the incidence of metastases to level IIb is rather low in OSCC, it is increasingly suggested that dissection of level IIb be avoided. In a meta-analysis by Lea et al., the overall pooled incidence of level IIB metastases from oral cancers was 6.02% (95 CI 3.45–8.59)^23^. Paleri et al. noted that overall incidence of metastatic disease to sublevel IIb in the N0 neck from any site was 2%, with an incidence of 3.9% from oral cancers, the incidence of isolated neck metastases to this level being only 0.3%^24^. We found similar results in the present study, with an overall incidence of 3.3% at level IIB. Despite such results and the risk of shoulder morbidity, studies have failed to clear the air concerning this issue, primarily due to lack of definitive evidence regarding oncological safety in avoiding submuscular recess dissection^25,26^. In various other published studies, authors seem reluctant to reach a conclusion on the necessity of level IIb dissection, only noting that the issue merits further discussion^23,27–29^. This uncertainty has led researchers to seek clinical criteria for level IIb dissection based on risk factors to level IIb metastasis.

To establish a customized neck dissection protocol, the present study developed a model using clinico-pathological parameters to predict the nodal status of level IIb. Based on the model, level IIa metastasis and LVP were predictors of level IIb metastasis. A majority of studies concur that positive nodes at level IIa predict level IIb metastases^9,30^. However, to the best of our knowledge, this is the first report of an independent association between LVP and level IIb metastasis in an OSCC patient cohort. Our predictive model indicates that the combined risk factors of level IIa metastasis and LVP significantly raise the level IIb metastasis rate. Our OSCC patient cohort comprises patients of all stages. Regarding unpredictable aspects of developed stage (III–IV) patients, our model exhibits comprehensive and decent performance, with an c-index(Harell’s) of 0.697 and model accuracy of 74.1%.

The prognostic importance of LVP in other solid tumors is well established and has been correlated with the development of distant metastases and survival^31–33^. LVP has been reported as a predictor for several well-known malignancies such as cervical, colorectal, and esophageal carcinomas^34,35^. It is also known to be associated with a high frequency of lymph node metastasis at the time of diagnosis and locoregional and distant metastatic recurrence in gastrointestinal and breast cancers^36,37^. Certain studies have emphasized preoperative assessment of LVP status in the therapeutic planning of breast cancers, noting that LVP status could help determine the need for sentinel lymph node biopsy and aggressive axillary lymph node dissections^38,39^. With regard to oral carcinoma, however, LVP does not appear to have drawn much attention^40^. Some studies have focused on LVP as an indicator of aggressive OSCC behavior along with perineural invasion (PNI); however, PNI was regarded as a more sensitive prognostic independent variable based on multivariate analysis^41–43^. It was only recently that a large OSCC patient cohort retrospective study emphasized lymphatic invasion (LI) and vascular invasion (VI) as independent risk factors in survival and recurrence of OSCC patients^44^. LVP is defined as groups of tumor cells attached to the lymphovascular vessel wall and projecting into the lumen of vessels in the capsule or outside the tumor. Although current guidelines do not incorporate LVP as independent indication for adjuvant radiotherapy (RT) or chemotherapy (CRT), strong consideration of adjuvant treatment seems adequate for patients with biopsy-proven LVP^45^. This is supported by a study of early OSCC wherein LVP was an independent risk factor for recurrence^46^.

In order for the model to have predictive value and offer clinical criteria for surgical modality, however, it is important that risk factors be confirmed pre-operatively. Various methods of confirming one of our risk factors, level IIa metastasis, are well known, including preoperative assessment by clinico-radiological modalities and intraoperative diagnosis by use of frozen section. Moreover, sentinel node biopsy (SNB) is a well-established technique that allows the surgeon to identify and excise the upper echelon nodes that are subclinical. This technique, which has the potential to select patients for selective neck dissections, has an excellent safety profile and high sensitivity^30^. Pre-operative confirmation of LVP, however, is rarely practiced, main specimen analysis usually being done post-operatively. We would thus like to suggest possible methods of pre-operatively confirming LVP. The first and simplest choice is an incisional biopsy which includes sufficient peri-tumoral tissue for a pathologist to examine. Lympho-vascular permeation being a submucosal infiltration, it is vital to include the submucosal portion, with its lympho-vascular network; specimens must be obtained from several spots surrounding the tumor whenever possible to increase the probability of detecting LVP. Moreover, topological characteristic of the oral cavity must be taken into consideration because the thickness and width of submucosa varies along different sectors of the oral cavity. Algaba suggested that reliable vessel identification is possible using the hematoxylin and eosin (H&E) method provided that the observation is rigorous and all tested cases are subsequently confirmed by means of CD34^31^. For difficult cases in which vessels are unidentifiable, an immunohistochemical procedure can be applied for ultimate confirmation^47^. However, pre-operative incisional biopsy may not always provide enough information regarding peritumoral tissue and LVP status. Another method for pre-operative confirmation of LVP is MRI-based radiomics, whose medical images provide information about many quantitative features via high-throughput analysis, in which descriptive or predictive models of tumor characteristics are constructed based on selected features^48,49^. A prediction model that incorporated both the radiomics signature and MRI axial lymph node status successfully predicted LVP status with an AUC of 0.763 in breast cancer^50^. To the best of our knowledge, radiomics has not been actively applied in the field of OSCC. Further studies on radiomics and risk factors of OSCC are anticipated, not only with regard to predicting LVP status but also in predicting other pathologically ominous features that have been nearly undetectable pre-operatively.

There are myriads of reasons other researchers have been cautious about omitting level IIb dissection. First of all, Robbins proposed the classification of levels of neck to facilitate inter-institutional communication among surgeons, oncologists, and diagnostic radiologists abroad^10^. The levels were defined according to anatomic landmarks instead of oncologic criteria or actual lymphatic flow. Lymph nodes of level IIa and IIb are part of the spinal accessory lymphatic and internal jugular lymphatic chains. The uppermost lymph nodes of the spinal accessory lymphatic chain overlap with the highest nodes of the internal jugular chain^51^. Omitting parts of the nodes of the same lymphatic chain based on arbitrary anatomic landmarks does not seem reasonable from a therapeutic or oncologic standpoint. Secondly, Oikawa et al noted that all subsites of oral cancer may develop retropharyngeal lymph node metastasis and that although rare, level IIb could constitute the first echelon node due to metastatic spread via the retropharyngeal lymphatic pathway^52,53^. Thirdly, many studies emphasizing oncologic safety without dissecting level IIb seem to have overlooked the presence of micro-metastasis (MM) or isolated tumour cells (ITC) in the pN0 nodes^54^. In a recent study, the rate of MM was as high as 9% in pN0 cases, the risk being highest in level IB and IIA^55^. Moreover, current pre-operative diagnostics to detect nodal micro-metastasis are not definitive. To mention complications concerning the spinal accessory nerve, level IIb dissection cannot be solely blamed for shoulder syndrome. Cervical nerves distributed along cervical level II–IV may also contribute to innervation of the trapezius muscle in up to 39% of cases^56^. Some authors have accordingly proposed that communications between cervical nerves and spinal accessory are responsible for remaining trapezius muscle motor function even after radical neck dissection^57^. Lastly, treatment modalities of OSCC, especially in managing the neck, are primarily based on TNM staging^9^. Our common recognition on oral malignancy is that increasing T stage has a close to linear correlation with regional and distant metastasis. However, recent research on the mechanisms of metastasis reveal that early T stage cancers have high metastatic potential^58^. There are certainly myriads of unveiled mechanism concerning cancer metastasis and proliferation and hasty generalizations regarding oncologic safety based on possibly loose statistics must be avoided.

Our study has several limitations. First, as a retrospective study, it may need further evaluation through a prospective study involving randomization in multiple centers. Second, a subgroup analysis including shoulder dysfunction would have increased the validity of our study. Thirdly, including clinically correlated numerical variables such as serum biomarkers would have maximized concreteness of predictive model and nomogram. Finally, although our predictive model was tested in a separate validation group and its fitness was confirmed, validation of our model with external data would have strengthened our findings. However, discovering LVP as prognostic factor of level IIb metastasis is fresh approach which can ignite further studies by supplementing aforementioned limitations.

## Methods

### Study participants

Our present study reviewed and collected medical records of patients who had undergone surgical treatment of OSCC at the Department of Oral and Maxillofacial Surgery, Yonsei University, from January 2000 to December 2020. Data of 541 biopsy-proven OSCC patients were obtained at this step. Patients with metastatic disease, secondary primary cancer, perioperative mortality, a history of previous radiotherapy or/and chemotherapy, or a history of previous head and neck cancer were excluded. The study group was further narrowed to OSCC patients who had undergone at least a unilateral selective lateral compartment dissection. The extent of neck dissection was determined according to the site, size and extent of the primary tumor and clinico-radiologic examination, including physical examination, as well as imaging techniques such as CT scanning, magnetic resonance (MRI) and PET-CT. The selective neck dissections (SNDs) performed included level IIb in all patients. A unilateral neck dissection was performed unless there were metastases in both sides of the neck, the primary tumor crossed the midline, or when tumors arose from the base of the tongue or floor of mouth. All surgical specimens were post-operatively sub-dissected according to sublevels and sent to the Department of Oral Pathology for evaluation. Every neck dissection was performed according to the anatomical and surgical boundaries suggested by Robbins et al.^15^. Patients with following adverse post-operative risk features were considered for adjuvant chemo-radiotherapy: pN positive, positive margin, perineural infiltration, vascular embolization, perineural infiltration, lymphovascular permeation and huge pathologic T3 or T4 lesions. Post-operative patient surveillance policy of our institute includes follow-up visits every 3 months for the first year, 2–6 months for the second year and every 4–8 months for the third to fifth year and finally annual visits after the fifth year. After excluding 182 for insufficient data (44) or meeting the exclusion criteria (138; patients treated without neck dissection), a total of 359 patients’ data were suitable for analysis. The study was approved by the Ethics Review Board of Yonsei University Dental Hospital Institutional Review Board (IRB No. 2-2020-0036). All research was performed in accordance with guidelines proposed in Declaration of Helsinki. However, Written or verbal informed consent was not obtained from any participants because Ethics Review Board of Yonsei University Dental Hospital Institutional Review Board waived the need for individual informed consent, as this study had a non-interventional retrospective design and all data were analyzed anonymously.

### Statistical analysis

The statistical analysis was performed using SAS V9.4 (SAS Institute., Cary NC, USA) and R package, version 4.0.4 (R Project for Statistical Computing, Vienna, Austria). Baseline differences between the training set and validation set were assessed using the Independent two sample t-test or Mann–Whitney U test for continuous variables and Chi-square test and Fisher’s exact test for categorical variables. Potential clinico-pathologic risk factors associated with level IIb lymph node metastasis in the 359-patient neck dissection group were assessed by logistic regression test.

Survival curves were plotted to estimate the prognostic effect of LVP and level IIa metastasis on overall survival and recurrence using Cox proportional hazard regression.

### Modelling process

Prior to building a prediction model, we split the data into two mutually exclusive sets. Approximately 65% of the overall dataset was designated the training set and was utilized to generate the prediction model. The remaining 35% of the data constituted the validation set, for use in estimating the model’s accuracy. Harrell’s c-index was used to elucidate the performance of the proposed method. Model fitness was verified via Chi-square test.
